# For Mothers

**Published:** 1872-12

**Authors:** 


### FOR MOTHERS.

Half of all the children born, die before their tenth year, nine tenths of them unnecessarily. Let mothers, therefore, bear in mind that the great safeguards of their little ones are: —

First. Keep them dressed abundantly warm all the time.

Second. Feed them at regular hours, and never an atom on any pretense, between times.

Third. Give them abundant sleep at regular times, and allow it at no others; never in the afternoon, after they are six months old; but never leave a child to go to sleep in fear, or with hurt feelings.

